# Strategic communication for NCD prevention: Enhancing visibility, engagement and policy action in Europe

**DOI:** 10.1177/14034948251384432

**Published:** 2025-11-07

**Authors:** Sólveig Karlsdóttir, Anita T. Munch, Hugrún Snorradóttir

**Affiliations:** 1 The Icelandic Directorate of Health, Reykjavik, Iceland; 2 The Norwegian Directorate of Health, Oslo, Norway

**Keywords:** Communication, dissemination, JA PreventNCD, inclusivity, communication strategy

## Abstract

**Background:**

JA PreventNCD represents a collaborative European Union initiative aimed at reducing the burden of non-communicable diseases (NCDs) across Europe by targeting common risk factors and addressing health inequalities.

**Aims:**

This article outlines the comprehensive communication and dissemination work developed to enhance the Joint Action on Cancer and other NCDs prevention: Action on Health Determinants (JA PreventNCD) project’s visibility, impact and stakeholder engagement.

**Methods:**

The communication and dissemination of the project is designed to be both strategic and inclusive. The approach centres on key objectives: supporting the JA PreventNCD goals of shaping future policies and advancing the prevention of NCDs by addressing communication challenges; increasing awareness; and enhancing stakeholder involvement. Using a mix of traditional and digital platforms, including press releases, newsletters, policy briefs, scientific publications, social media and interactive events, the strategy facilitates targeted engagement with policymakers, healthcare professionals, non-government organisations and the public. A strong emphasis on collaboration while recognising each country’s unique challenges and opportunities in communicating NCD prevention.

**Conclusions:**

**By adhering to principles of inclusivity and accessibility, the communication work strives to engage diverse audiences effectively, encourage dialogue, support policy change and set the agenda. Ongoing evaluation measures ensure that the approach remains responsive, evidence based and impactful, aligning closely with the overarching goals of JA PreventNCD.**

## Introduction

Non-communicable diseases (NCDs) are the leading cause of mortality and morbidity in Europe, responsible for 90% of deaths annually [1]. The COVID-19 pandemic has further exacerbated these challenges, delaying diagnosis and treatment, increasing mental health issues and widening health inequities across socioeconomic and demographic groups [2,3]. Despite the scale of the problem, much of the disease burden is preventable. The COVID-19 pandemic also underscored the critical need for integrating targeted communication with decision-making processes and ensuring that messages were accessible to all, regardless of health literacy, language skills, digital skills and social economic status [4]. Effective communication strategies that prioritise inclusivity and clarity can significantly enhance public understanding and compliance during health crises [5].

The Joint Action on Cancer and other NCDs prevention: Action on Health Determinants (JA PreventNCD) project is a key component of the European Union (EU)’s health initiatives, aligning with Europe’s Beating Cancer Plan and the Healthier Together Initiative to combat NCDs [6]. These frameworks highlight prevention, early intervention and cross-sectoral collaboration to address shared risk factors and social determinants of health. Through coordinated, evidence-based actions, JA PreventNCD supports the EU’s goal of reducing the NCD burden and promoting equity across member states. The European Commission sees communication as a cornerstone of strengthening collaboration, sharing best practices and raising public awareness about health priorities [7]. By prioritising accuracy and reliability, the Commission aims to combat misinformation and promote evidence-based messaging [8].

Effective communication plays a crucial role in shaping understanding and fostering collaboration across various sectors. It can enhance social cohesion, improve health outcomes and drive meaningful policy change [9]. Within the JA PreventNCD effective communication and dissemination are pivotal to its success, ensuring that the project’s objectives, activities and outcomes are shared, understood and utilised by the project partners and stakeholders. Strategic dissemination not only facilitates knowledge transfer, it can also inspire policy action and public engagement, strengthening collective commitment to the prevention of NCDs [10]. Given the diverse range of stakeholders including policymakers, academics, healthcare professionals and the public, targeted communication and dissemination efforts are essential to address challenges and drive meaningful, lasting impact.

The communication and dissemination work within JA PreventNCD is built on a communication and dissemination strategy. These following key approaches are used to effectively broadcast the information produced within the Joint Action. Communication engages target audiences such as the public, stakeholders and policymakers to raise awareness about the project and its impact. Dissemination additionally involves sharing project results with the scientific community. Both activities help ensuring that the project’s achievements reach a broad audience and contribute to scientific and societal advancements [6]. Targeted, clear and inclusive messaging presented in a simple, visually understandable format ensures that key activities and results effectively reach diverse target audiences, fostering equity and diversity [5,11,12].

However, the communication and dissemination work face various challenges. First, health promotion and NCD prevention efforts often lack political support due to their long-term nature and benefits that are hard to measure compared with medical treatments. Prioritising health promotion when resources are scarce requires strong political will and courage. Budget silos and competing priorities hinder investments in holistic health approaches [5]. Second, the tobacco, alcohol and food industries are formidable opponents for governmental health promotion work, as the World Health Organization *Commercial determinants of health* report points out [1]. These industries’ marketing budgets and lobbying resources undermine health promotion efforts. Third, there is growing concern that documented knowledge is losing authority, as self-proclaimed experts, often driven by various agendas, gain visibility and are increasingly seen as equal to established expert voices in social and traditional media. Internet algorithms reward controversial content, and studies are misused for commercial gain [13,14]. This is not specific to health information, but the consequences can be particularly dramatic for those with low health literacy levels [15]. Lastly, differences in language, culture, political priorities and health literacy levels among the EU member states can create barriers to effective communication. The scale of the EU’s governance structure and population is large, so communication and dissemination work face significant obstacles [16].

This article outlines the framework for the communication and dissemination work executed in the JA PreventNCD project. It explores the methods, tools and strategy that guide the project’s efforts to engage stakeholders, increase visibility and address barriers and possible limitations to effective communication. By aligning with the project’s overarching goals and ensuring effective, inclusive and adaptable dissemination activities, JA PreventNCD aims to amplify its impact on sustainable health improvements and promote health equity across Europe.

## Methods

This chapter highlights the communication and dissemination work within the JA PreventNCD project, including branding, stakeholder engagement, communication and dissemination strategy and targeted outreach.

### Branding

**Figure 1.**
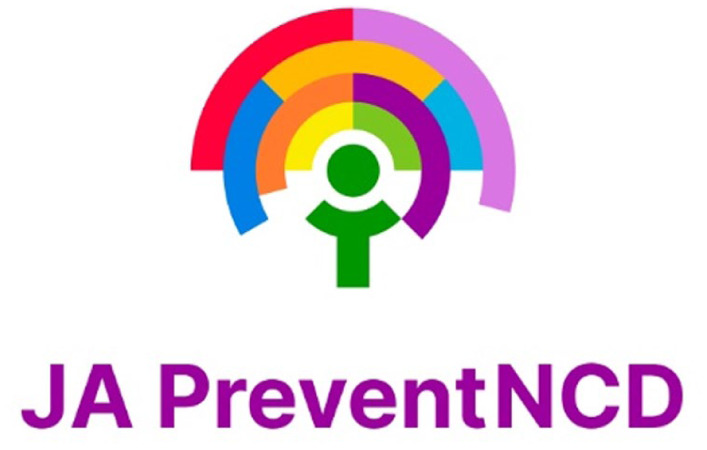
JA PreventNCD logo. JA PreventNCD: Joint Action on Cancer and other Non-communicable Diseases prevention: Action on Health Determinants.

Branding is important, as it establishes a clear identity and enhances recognition and engagement among stakeholders [17]. Consistent branding facilitates better understanding of the project’s goals, making it easier for partners, funders and the public to engage with and support the initiative [18]. The logo of JA PreventNCD is distinguished by a human figure set against a stylised sunrise. This figure denotes community and solidarity, and the sunrise, which is creatively based on a doughnut chart, underscores the project’s dedication to health and wellbeing, symbolising both new beginnings and the scientific rigour that underpins all outputs (see Figure 1). The logo is inspired by the Dahlgren–Whitehead rainbow model for determining health inequalities and the United Nations Sustainable Development Goals [19].

### Stakeholder analysis

**Figure 2.**
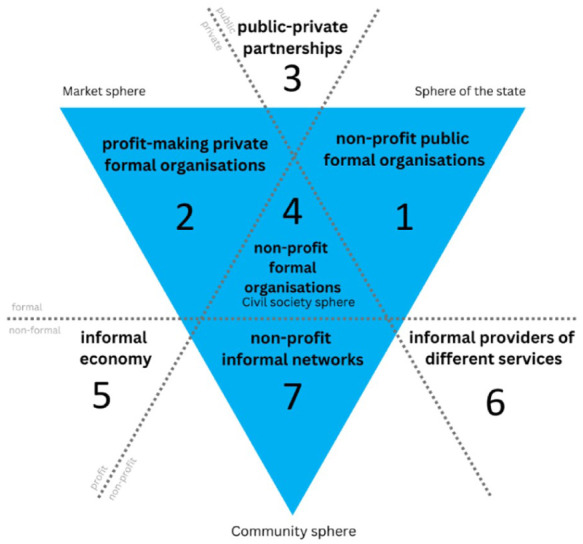
Welfare triangle (adapted from Pushkarev et al. [22]).

The goal with the stakeholder analysis is to cast a wide net, ensuring representation not only from the mainstream stakeholders but also from those who are often marginalised or overlooked. To do so, the stakeholders will be identified by the welfare triangle based on the ‘welfare mix’, (see Figure 2) a concept that was developed to enable identification of differences among groups of welfare states [20,21]. The welfare mix has been adapted and expanded to aid understanding of how different spheres of society, such as the state, market and civil society contribute to welfare provision in varying contexts. This approach has been effectively applied in previous Joint Actions, to support stakeholder analysis [22]. For the purposes of the JA PreventNCD, the welfare triangle is used to identify as many relevant stakeholders as possible. By employing a structured approach, key stakeholders across various sectors and communities can be identified, enriching the project’s participants understanding of the stakeholders and engagement strategies. This approach increases the likelihood of targeting the right audience with relevant information [23].

Furthermore, it is important to actively leverage synergies and strengthen collaboration with other EU health initiatives, such as the Joint Action on Cardiovascular Diseases and Diabetes, Joint Action Mental Health Together (MENTOR) and international organisations, such as the World Health Organization. Leveraging relationships with them strengthen the communication and dissemination efforts of the Joint Action.

### Communication and dissemination strategy

The JA PreventNCD Communication and Dissemination Strategy was designed as a tool to support the overall goals and objectives of the JA PreventNCD project (see Figure 4). It ensures targeted, inclusive, effective and sustainable communication of the project’s objectives, activities, results, deliverables and aims to engage all relevant stakeholders, including project partners, policymakers, healthcare professionals, patient and user organisations, non-government organisations (NGOs), academia and the public.

**Figure 3.**
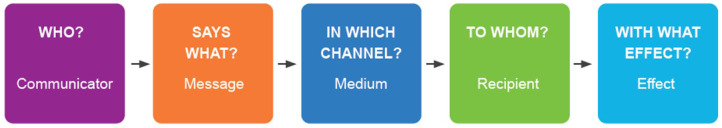
Laswell’s model of communication (adapted from Laswell [24]).

Its primary objectives include raising awareness about the societal burden of NCDs, enhancing stakeholder engagement, inspiring action and supporting the implementation of evidence-based prevention measures across Europe. Through clear, inclusive and culturally sensitive messaging, the strategy strives to reduce health inequities and promote the adoption of sustainable policies and behaviours. By applying Lasswell’s communication model, the strategy addresses the essential elements of effective communication: *Who communicates? What is communicated? To whom? Through which channels? And with what effect?* (see Figure 3). This structured approach ensures clarity and precision, allowing messages to resonate deeply with diverse target audiences, mentioned above [24,25].

**Figure 4.**
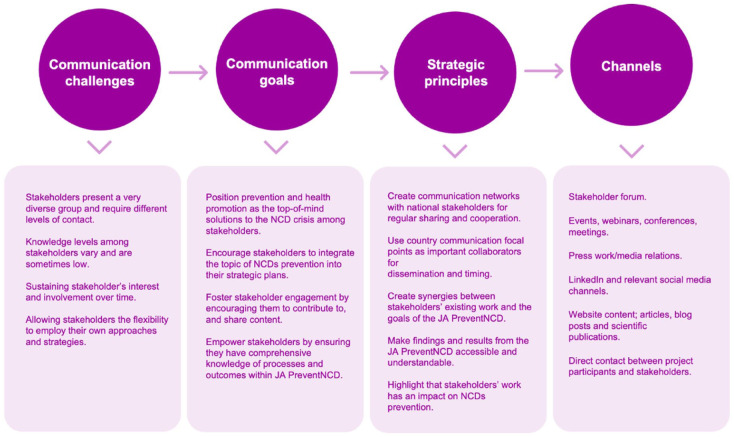
Overview of the JA PreventNCD strategic communication principles. JA PreventNCD: Joint Action on Cancer and other Non-communicable Diseases prevention: Action on Health Determinants.

The JA PreventNCD Communication and Dissemination Strategy’s focus is on a wide range of target audiences. Policymakers are engaged through policy briefs and direct consultations, while the public is reached via accessible campaigns promoting healthy behaviours. Stakeholders such as NGOs and healthcare professionals are actively involved in collaborative initiatives, strengthening their role in disseminating project outputs.

At the same time, in the Communication and Dissemination Strategy, potential challenges and barriers have been identified that could hinder the project’s impact. These include a lack of political interest, will or courage, stemming from the long-term nature of prevention efforts and the inherent difficulty in measuring their immediate benefits [26]. In many instances, investments in health promotion are deprioritised in favour of initiatives that promise quicker, more tangible returns, especially when budgets are siloed across various sectors [27]. Lack of trust in documented knowledge, misinformation, fake news and information overload. These factors complicate communication efforts as a growing number of self-proclaimed experts distort scientific information for personal or commercial gain, particularly in health-related areas [13,14]. And lastly, industry influence and lobbying, particularly from sectors like tobacco, alcohol and food pose a challenge with their substantial marketing budgets and lobbying resources. These industries continue to use substantial resources to oppose public health measures, undermining governmental health promotion efforts [1].

At its core, the strategy aims to raise awareness of NCD prevention, reduce health inequities, inspire behavioural and policy change and promote collaborative efforts across sectors. Tailored communication approaches play a critical role in achieving these objectives. For instance, messages are customised based on the audience’s cultural and political contexts, varying levels of health literacy and readiness to act. These tailored strategies are implemented through an array of tools, including policy briefs, scientific publications, social media campaigns, workshops, events and webinars, which are designed to maximise outreach and engagement. Through its comprehensive and adaptable framework, the Communication and Dissemination Strategy exemplifies how strategic communication can contribute to meaningful change at societal level and through policy change.

### Multi-level approach to communication

A key component of the strategy is its multi-level approach, which ensures alignment and consistency across the project. Communication focal points from each work package and national-level representatives from the leading institutions in participating countries play a vital role in engaging key stakeholders and disseminating project findings. By working closely with the lead institutions in each participating country, the strategy ensures that communication is adapted to national contexts while maintaining coherence across the project. The focal points, both at the work package and national levels, create their own communication strategies that align with the overarching project strategy, ensuring coherence while adapting to specific national contexts [4]. Moreover, these focal points facilitate cross-sectoral dialogue, ensuring that evidence-based prevention measures reach decision-makers and other key stakeholders. Recognising the diversity of languages and cultural contexts across Europe, in collaboration with national focal point, materials and messages are adapted to reflect cultural nuances, ensuring that the messaging is both relevant and understandable across different communities [5,12].

### Ensuring consistency through a common glossary

To further enhance clarity and alignment across the project, the JA Glossary serves as a foundational tool for communication. Developed to ensure a shared understanding of key terms and concepts for all project participants, as well as external audiences. This is particularly important in a multinational initiative, where terminology can have different interpretations depending on context. The glossary focuses on standardising key terms, particularly those used across multiple work packages and those with potentially varied contextual meanings. For example, message framing, an approach in health communication that emphasises either the benefits (gain framed) or the risks (loss framed) of specific health behaviours, is one such concept where consistent terminology is crucial to ensure clear, persuasive and culturally sensitive messaging across diverse contexts [28]. Additionally, it provides guidance on using inclusive language, to ensure that our communication reaches a broader audience and builds trust [29].

### Website and social media

The JA PreventNCD website, www.preventncd.eu, is a cornerstone for achieving the project’s communication and dissemination objectives. The website serves as the main hub for information and interaction, offering updates on the Joint Action’s objectives, results, deliverables and synergies. It features news, blogs, articles, project outputs, event listings, partner details and resources such as webinar recordings and other materials. The website is connected to Google Analytics, providing insights into online performance and enabling the project team to refine strategies and content to increase visitor numbers and engagement. In addition to its standalone role, the website is linked to the project’s social media platforms, LinkedIn, Facebook, YouTube and newsletters, forming a cohesive and dynamic communication ecosystem. Each social media platform enhances the project’s communication strategy by targeting specific audiences and content types [30].

### Scientific dissemination

Scientific dissemination within the JA PreventNCD project is grounded in the principles of open science, ensuring that knowledge and results are accessible to all and free of charge. The primary aim of this dissemination is to maximise the impact of the work conducted under the Joint Action, facilitate progress for other researchers and contribute to the advancement of scientific knowledge. By making these results publicly available, the project aims to foster collaboration and share the outcomes of European research. As part of this effort, JA PreventNCD has set a target of publishing 50 scientific articles and 60 policy briefs throughout the 4-year duration of the project. This openness ensures that the results of JA PreventNCD reach the appropriate audiences and are used to inform policy, research and practice across Europe [8].

### Webinars

Monthly webinars, both internal and external, take place to enhance the impact of the project by setting the agenda and influencing key target audiences and even the public. They serve as a platform for increasing visibility and awareness of JA PreventNCD. Through the sharing of research findings, discussions on key topics and promotion of best practices, the webinars aim to foster interdisciplinary collaboration and drive engagement across sectors. Furthermore, in a consortium of this capacity, building a strong project community is also an important aspect of communication.

### Evaluation of the communication and dissemination work

The evaluation of communication and dissemination activities within the JA PreventNCD are carried out through ongoing monitoring to assess their effectiveness and identify areas for improvement. This approach is supported by regular dissemination reports, in line with both internal and technical reporting to the EU. Throughout the project, two official communication and dissemination reports will be published, to assess progress and outcomes.

The evaluation process involves comprehensive monitoring of various channels and activities. This includes tracking website performance and social media engagement, to assess the reach and impact of our communication efforts. As part of the output indicators for the Joint Action the website traffic is analysed to gain insights into user engagement, audience demographics and the effectiveness of specific content. In addition, social media platforms are monitored for engagement metrics, helping to understand how well the messages resonate with target audiences. The goal of the evaluation and monitoring is not only to measure the effectiveness of the communication efforts but also to identify opportunities for improvement and ensure the right audiences are reached.

## Discussion

The EU highlights the importance of effective communication to ensure the visibility and impact of EU-funded projects. Clear and reliable communication is essential to combat misinformation, enhance collaboration and raise public awareness of health priorities. To enhance the impact of the project, the communication and dissemination work algins with the EU presidency of member states. This alignment will leverage the heightened profile and media coverage that comes with the presidency.

In addressing the communication and dissemination challenges, the JA PreventNCD project emphasises the critical role of strategic communication and cross-sectoral collaboration in advancing NCD prevention across Europe. Tackling these challenges requires a comprehensive and evidence-based approach, informed by the strategic measures outlined in the project. Effective communication aims to ensure that the project’s messages reach the right audiences by using the welfare mix, empowering stakeholders to take action and support long-term health improvements across Europe.

Key targets of the project are the scientific publications and policy briefs, which are not only a measure of the project’s output but also serve as indicators of its broader impact. Achieving these targets will allow assessment of the project’s reach and influence within the scientific community, policy circles and public health sectors. Publications and policy briefs are critical tools for translating scientific findings into actionable knowledge, shaping policy and informing practice. Each publication or brief will be an opportunity to disseminate the project’s outcomes to key stakeholders, influence decision making and ensure the sustainability of its impact.

The participation of trusted entities in participating countries is furthermore critical to the success of JA PreventNCD. These entities, which hold the confidence of the public, bring credibility and legitimacy to the project’s goals and activities. Their involvement fosters trust among diverse stakeholders, enhances receptivity to the project’s outcomes and contributes to the impact resonating at both local and broader levels. This trust is not only essential for effective dissemination but is also a key determinant of the project’s long-term success and sustainability.

Large projects like JA PreventNCD face a variety of challenges that can hinder the effectiveness of communication and dissemination efforts. Siloed thinking is a common risk in such extensive projects, which can limit cross-cutting communication. To address this, the project actively promotes collaboration across work packages, ensuring that communication is integrated throughout the entire project. By aligning key messages across various topics within the Joint Action the project enhances its impact and encourages broader engagement.

While building on the insights gained from previous EU-funded projects, JA PreventNCD recognises that a project of this scale and scope provides an opportunity to elevate communication efforts to a more structured and impactful level. The Joint Action offers a unique opportunity to strengthen and formalise communication strategies, benefiting not only JA PreventNCD but also, hopefully, future Joint Actions. By formalising communication practices, the project lays a foundation for future initiatives to build upon and further advance these strategies.

## Conclusion

Effective communication and dissemination are important pillars in achieving the goals of the JA PreventNCD project. By adopting a strategic, inclusive and evidence-based approach, the project ensures its messages reach diverse audiences, fosters cross-sectoral collaboration and recommends policies to enhance health and wellbeing for all. Despite notable challenges, such as possible lack of political will, industry influence and the spread of misinformation, the project’s structured communication efforts and stakeholder engagement aim to provide a foundation for lasting impact.
